# The Impact of Calling on Employee Creativity: Evidence From Internet Companies

**DOI:** 10.3389/fpsyg.2021.773667

**Published:** 2021-11-25

**Authors:** Jia Lv, Wanming Chen, Yufang Ruan

**Affiliations:** ^1^College of Economics and Management, Nanjing University of Aeronautics & Astronautics, Nanjing, China; ^2^School of Economics and Management, Nantong University, Nantong, China; ^3^School of Management, Shanghai University, Shanghai, China

**Keywords:** calling, career commitment, responsible leadership, creativity, internet companies

## Abstract

With the rapid development of technology and increasingly fierce competition in the global market, innovation has become the most important competitive advantage for enterprises. Employee creativity is widely considered the source of organizational innovation. This study explores the antecedents of employee creativity from the perspective of career development in the context of high-technology industry. Specifically, we examine the effects of calling on employee creativity through the mediation of career commitment and the moderation of responsible leadership. With data collected from a sample of 218 respondents from internet companies, a series of regression analyses was conducted to test the proposed hypotheses. In addition, a moderated mediation model was further examined. Discussion, implications, and limitations are presented.

## Introduction

Economic globalization and technological innovation are the themes of today's world. In particular, information technology enables people to share and spread resources conveniently and rapidly, making competition among organizations increasingly fierce. Almost all enterprises, particularly high-technology companies, emphasize the importance of human capital. Faced with a complex and changing external environment, employee creativity has become the driving force and core competitive capability of organizations.

Under these circumstances, the question of how to help high-technology enterprises improve their innovativeness to gain competitive advantages attracts both business scholars and practitioners. Organizational innovativeness depends on employees' creativity. Therefore, this research hopes to identify the factors enhancing employee creativity in the high-technology industry and to explore its mechanism.

In the domain of vocational research, an important concept is calling, which refers to the deep meaning people feel toward their work (Wrzesniewski, 2003). It relates to self-fulfillment, meaningfulness, and happiness in one's career choice and development. According to Duffy and Dik (2013); calling is salient in both university student and adult populations, and it can enhance career, commitment, career maturity, work meaning, job satisfaction, life meaning, and satisfaction levels. Dobrow and Tosti-Kharas (2011) identify the positive effects of calling on career-related efficacy, the professional pursuit of the calling domain, and the satisfaction with the calling domain. These studies show that employees who have a strong sense of calling are likely to pursue superiority in their work. These employees realize the meaningfulness of their work and tend to voluntarily invest more time and energy in it, demonstrating a high degree of career commitment. They will gain higher career achievements and job satisfaction from work and create greater value for their employers. Therefore, we postulate that in the high-technology industry, calling may inspire employees' innovativeness and creativity through the mediation of career commitment.

Previous studies have indicated that leadership style may affect various organizational behaviors (e.g., Cable and Judge, 2003; Benjamin and Flynn, 2006; Graham et al., 2015). Some studies have focused on employee innovative behavior. For example, Lei et al. (2011) discover that transactional and transformational leadership exert negative and positive influences on employee innovative behavior, respectively. Fang et al. (2019) find that inclusive leadership, which emphasizes people-oriented justice, and fairness, may stimulate the new generation of employees' innovative behavior through the mediation of social capital. However, no study has been conducted on the effects of responsible leadership on innovative behavior.

This study introduces responsible leadership as an adjustment variable, aiming to explore the effect of responsible leadership on employee creativity and its mechanism, as well as the moderating role of responsible leadership in the relationship between professional commitment and employee creativity. As a novel leadership style, responsible leadership is identified as a social–relational and ethical phenomenon. Different from the prevailing leadership style theory, which emphasizes the relationship between leaders and followers in an organization, responsible leadership exists in the interaction with various followers as stakeholders inside and outside a corporation (Maak and Pless, 2006). It focuses on value creation and social change, and it tends to satisfy the needs of various stakeholders while pursuing organizational goals. It advocates communication with stakeholders through open discussions and considers the feelings and values of others to obtain common interests. Responsible leadership provides an atmosphere that encourages employees with a high sense of calling to work creatively. Therefore, we postulate that responsible leadership may play a moderating role in the relationship between calling and employee creativity.

## Calling and Employee creativity

Work as a calling was originally a religious concept, but it has become a popular concept across psychological disciplines in recent years. Calling refers to a psychological state that reflects individuals' passion for a job that is considered the meaning of their life (Duffy and Dik, 2013). It reflects individuals' attitudes and perceptions toward their current work (Dobrow and Tosti-Kharas, 2011). Many empirical studies have examined the consequences of calling in work performance and quality of life. For example, Duffy and Sedlacek (2007) examine the effect of calling on the career development of American college students. They find that students' sense of calling is positively correlated with decidedness, comfort, self-clarity, and choice–work salience and negatively correlated with indecisiveness and lack of educational information. Hirschi and Herrmann (2012) find that calling is positively correlated with college students' decidedness and self-efficacy. Dobrow and Tosti-Kharas (2011) find that calling promotes individuals' work engagement and satisfaction, career-related self-efficacy, and clarity of professional identity. Hirschi and Herrmann (2013) report that the presence of a calling can affect life satisfaction through the mediation of vocational identity achievement among German university students. Duffy et al. (2012) discover that university students' sense of calling affects life satisfaction with the moderation of core self-evaluation and the mediation of personal meaning in life and academic satisfaction.

Calling has also been examined in Eastern societies. Based on a qualitative study among Chinese college students, Zhang et al. (2015) derive a multi-dimensional construct of calling. This construct has four dimensions— career-related calling, guiding force, meaning and purpose, altruism, and active tendency—which are considered to greatly converge with those found in Western cultures. Using a sample of Korean salespersons, Park et al. (2015) find that the sense of calling affects organizational citizenship behavior through the mediation of occupational self-efficacy in Korea.

Employee creativity refers to the ability to generate novel things or ideas that can lead to new products, services, production methods, or work processes (Amabile and Gryskiewicz, 1989). Both personality traits and organizational context can stimulate an individual's creativity. McCrae and Costa (1997) maintain that individuals with high openness to experience are more likely to absorb and integrate new information and seek novel environments and experiences. Amabile and Gryskiewicz (1989) identify several environmental stimulants to creativity, including freedom, challenge, resources, and supervisor, and some obstacles to employment creativity, such as time pressure, politics, status quo, and evaluation. Amabile et al. (1996) find that the perceived work environments of group supports, challenging work, and organizational and supervisory encouragement can enhance employee creativity. Through multi-level studies, Liu et al. (2011) discover that employees can translate organizational autonomy support and individual autonomy orientation to individual job creativity through harmonious passion.

Calling can improve work performance. For high-technology companies, innovativeness and creativity are the most important occupational characteristics. From the perspective of social information processing, employees with a high sense of calling often perceive the significance of their work for the organization, enhancing intrinsic motivation toward individual creativity (Amabile, 1994). Moreover, calling helps motivate employees to make great efforts to absorb new knowledge and master advanced skills, enabling them to better deal with challenges and enhance their creativity. Therefore, we propose a positive relationship between calling and employee creativity.

H1: Calling is positively related to employee creativity.

### Career Commitment as a Mediator

Career commitment reflects individuals' attitudes toward their career and profession (Blau, 1985). It shows a certain expectation that employees have about their jobs. It is related to other work attitudes, such as work ethic endorsement, job involvement, and organizational commitment (Blau, 1988; Irving et al., 1997). This shows that individuals with a high career commitment tend to center their lives around work and vacation. They are likely to spend more time and energy to succeed in their careers (Meyer et al., 1993). The antecedents of career commitment are work–role salience, career satisfaction, and organizational opportunity for development (Aryee and Tan, 1992). Career commitment also affects work outcomes, such as job satisfaction, turnover intention, and career success (Irving et al., 1997; Goulet and Singh, 2002; Poon, 2004). Employees with a high career commitment recognize the value of their jobs, and they tend to make more efforts to improve their knowledge, skills, and performance. They more easily acquire positive emotions from their profession, are more likely to proactively pursue career success, and have lower turnover intentions.

In organizations, employees with high career commitment usually pay more attention to career development, set higher career goals, and make greater efforts to proactively develop and cultivate relevant career skills. Ellemers et al. (1998) suggest that career commitment indicates an individual's motivation to pursue career development and advancement. According to self-determination theory, career commitment acts as an intrinsic driving force to urge individuals to manage their careers (Savickas and Porfeli, 2012). When employees have a strong career commitment, they are always willing to make efforts to proactively create novel products, processes, and systems, especially in high-technology enterprises. Conversely, low career commitment usually indicates dissatisfaction with career and profession. These employees lack the motivation to seek further development in their careers and companies.

Based on the previous analysis, we can conceptually derive a close relationship between calling and career commitment. Specifically, individuals with a strong sense of calling can effectively improve their career self-efficacy and career planning, leading to stronger career commitment and work outputs. In an empirical study, Duffy et al. (2011) find the positive effects of the sense of calling on various work-related outcomes, including career commitment, organizational commitment, and job satisfaction. Among these variables, career commitment is identified as a mediator that links the calling–organizational commitment relationship to the calling–job satisfaction relationship. The authors note that career commitment may play a significant role in linking calling to work-related outcomes.

Moreover, employees with a high sense of calling can stimulate career commitment in their work and contribute to the development of an organization. On the contrary, employees who are weak in calling are often short of career commitment and unwilling to stimulate creativity.

H2: Career commitment mediates the relationship between calling and employee creativity.

### Responsible Leadership as a Moderator

Responsible leadership is defined as the ability to build, cultivate, and maintain mutual relationships with stakeholders inside and outside the organization, which emphasizes responsible behavior in cooperation. Responsible leadership aims to realize the business vision through sharing meaning (Maak and Pless, 2006; Maak, 2007). It integrates the theories of social responsibility and leadership.

Compared with other leadership styles, such as responsible leadership, transformational leadership, ethical leadership, and servant leadership, responsible leadership stresses value creation and social transformation. With the pursuit of organizational goals, it focuses on the demands of various stakeholders and seeks to engage in democratic communication with them. It requires leaders to take others' emotions and values into consideration to form common interests. Voegtlin (2011) finds that responsible leadership positively affects employees' job satisfaction. According to Voegtlin et al. (2012); a good relationship between responsible leaders and stakeholders may motivate them to share knowledge, information, and experiences, which can promote enterprises' innovative culture. In summary, responsible leadership has a positive effect on balancing and coordinating the internal and external relationships in an organization, creating an open and innovative environment and enhancing employee creativity.

Creativity is the source of innovation. All the innovative processes and products in an organization come from the deep and multi-dimensional development of creativity. If employees in the organization can continuously maintain creativity, they will be able to obtain quality resources and innovative opportunities in the market, develop more excellent products and service processes over competitors, and effectively improve organizations' advantageous position in the fierce competition. In the process of organizational innovation, leadership style is a significant factor: how leaders in the organization build relationships not only with subordinates but also with other stakeholders. Previous studies find transformational leadership (Shin and Jing, 2003; Gong et al., 2009), empowering leadership (Zhang and Kathryn, 2010), and ethical leadership (Feng et al., 2018; Javed et al., 2018; Mo et al., 2019) may exert influences on employee or team creativity.

This study focuses on responsible leadership and examines it under the framework of the leader–stakeholder interaction model. We believe that responsible leaders can motivate employees' inner work enthusiasm through self-consciousness and high ethical standards to make them work proactively. They listen to employees' opinions and give them transparent support. In addition, responsible leaders have rich social relations that enable them to demonstrate a high level of openness and trust toward subordinates. According to social exchange theory, transparent leadership behaviors contribute to high-quality leader–member exchange (LMX) relationships. In a longitudinal study, Volmer et al. (2012) find that a high-quality LMX, which is characterized by a great degree of freedom in work and decision making, facilitates innovative behaviors. Therefore, a perceived responsible leadership style can stimulate the relationship between career commitment and employee creativity.

H3: Responsible leadership moderates the relationship between career commitment and employee creativity such that the relationship is stronger for those with higher rather than lower perceived responsible leadership.

Based on H1–H3, we further propose a moderated mediation model— that is, responsible leadership plays a moderating role in the indirect influence of calling on employee creativity via career commitment.

H4: Responsible leadership moderates the indirect influence of calling on employee creativity via career commitment. Specifically, the indirect effect is stronger when responsible leadership is high rather than low.

## Methods

This study collected data through a survey of executives and staff in internet companies. A total of 230 respondents participated in the survey. With invalid questionnaires excluded, a total of 218 valid samples were obtained.

As shown in Table 1, among the valid sample, 142 were males (65.1%), 143 were under 35 years old (65.6%), most of the respondents had more than 10 years of working years (44%), and the majority had a bachelor's degree or above. The questionnaire survey was distributed in several major Chinese cities, including Shanghai, Beijing, and Xi'an.

**Table 1:** Demographic variables.

**Demographic variables**		**Percentage (%)**
Gender	Male	65.14
	Female	34.86
Age	Under 35	65.60
	Over 35 years old	34.40
Working years	1–3 years	19.70
	4–6 years	17.90
	7–9 years	18.30
	More than 10 years	44.00
Education level	PhD	6.90
	Master	40.80
	Undergraduate	35.80
	College	16.70

### Measurement

This study used established scales to measure calling, career commitment, responsible leadership, and employee creativity. Calling and career commitment were measured using twelve items adapted from Dobrow and Tosti-Kharas (2011) and seven items adapted from Suddaby et al. (2009). The reliability coefficients for the two scales were 0.958 and 0.922, respectively. Five items from Voegtlin (2011) were adopted to measure responsible leadership, with a Cronbach's α of 0.947. Three items from Baer (2012) were used to measure employee creativity, with a Cronbach's α of 0.818.

In addition, this study also considered some important demographic variables as control variables, including gender, age, working years, and education level.

### Data Analysis

R software was used to test the construct validity of the scales through confirmatory factor analysis. As shown in Table 2, compared with the three-factor, two-factor, and single-factor models, the four-factor model has the best model fit of χ^2^ = 550.693, df = 183, CFI = 0.916, IFI = 0.916, SRMR = 0.046. This shows that the four variables have good discriminant validity.

**Table 2:** Confirmatory factor analysis results.

**Model**	**χ^2^**	***df***	**χ^2^ /df**	**SRMR**	**CFI**	**NNFI**	**IFI**
Four factors: C, CC, RL, ER	550.693	183	3.01	0.046	0.916	0.903	0.916
Three factors: C+RL, CC, ER	1196.555	186	6.43	0.091	0.769	0.739	0.770
Two factors: C+RL, CC+ER	1396.114	188	7.43	0.107	0.724	0.691	0.725
Single factor: C+RL+CC+ER	1687.224	189	8.93	0.112	0.657	0.619	0.659

*C, Calling; CC, Career Commitment; RL, Responsible Leadership; ER, Employee Creativity*.

Table 3 presents the mean and standard deviation of each variable and the correlation coefficients between them. Calling and career commitment are found to be strongly correlated (r = 0.738, *p* < 0.01), calling and employee creativity are moderately correlated (r = 0.382, *p* < 0.01), and career commitment (r = 0.380, *p* < 0.01) and responsible leadership (R = 0.378, *p* < 0.01) are significantly positively correlated with employee creativity, respectively. This shows preliminary support for the proposed hypothesis.

**Table 3:** Variable mean, standard deviation and correlation coefficient table.

**Items**	**M**	**SD**	**1**	**2**	**3**	**4**	**5**	**6**	**7**
1. Age	3.270	0.758	–						
2. Gender	0.349	0.478	−0.143^*^	–					
3. Education level	3.350	0.910	0.019	−0.239^**^	–				
4. Working years	2.870	1.182	0.662^**^	0.025	−0.154^*^	–			
5.Calling	5.110	1.284	0.134^*^	−0.088	−0.061	0.080	–		
6. Career Commitment	5.524	1.158	0.058	−0.153^*^	0.022	0.004	0.738^**^	–	
7. Responsible leadership	5.296	1.304	0.008	−0.028	0.011	−0.010	0.621^**^	0.585^**^	–
8. Employee creativity	4.936	1.337	0.202^**^	−0.196^**^	−0.064	0.197^**^	0.382^**^	0.380^**^	0.378^**^

* *p < 0.05*,

** *p < 0.01*.

### Hypothesis Testing

This study adopted a multi-step regression method through SPSS 22.0 to examine the hypotheses. The regression results are shown in Table 4. In Model 4, after controlling for the demographic variables, calling has a significantly positive effect on employee creativity (β = 0.346, *p* < 0.001). Therefore, hypothesis 1 is supported.

**Table 4:** Main effects and mediation effects.

**Items**	**Career commitment model 1**	**Model 2**	**Creativity model 3**	**Model 4**	**Model 5**
Age	0.061	−0.041	0.089	0.041	0.050
Gender	−0.149^*^	−0.083	−0.209^**^	−0.178^**^	−0.160^*^
Education level	−0.020	0.045	−0.095	−0.065	−0.075
Working years	−0.036	−0.019	0.129	0.137	0.141
Calling		0.740^***^		0.346^***^	0.186^*^
Career commitment					0.216^*^
R2 value change	0.026	0.531	0.090	0.116	0.021
F value change	1.408	53.342^***^	5.281^***^	31.090^***^	5.642^*^

* *p < 0.05*,

**p *p < 0.01*,

*** *p < 0.001*.

This study followed the steps proposed by Baron and Kenny (1986) to test the mediating effect of career commitment between calling and employee creativity. Model 2 shows that calling has a significantly positive effect on career commitment (β = 0.74, *p* < 0.001). In Model 5, when the independent variable (calling) and the mediating variable (career commitment) are put into the regression model, career commitment is positively correlated with employee creativity (β = 0.216, *p* < 0.05), and the relationship between calling and employee creativity is significant (β = 0.186, *p* < 0.05). However, the regression coefficient decreases from 0.346 in Model 4 to 0.186 in Model 5, indicating that career commitment is a partial mediator in the relationship between calling and employee creativity. Therefore, hypothesis 2 is supported.

In order to further validate the mediating role of career commitment between calling and employee creativity, we used the SPSS Process macro to conduct the bootstrap test. The results show that the indirect effect value of career commitment is 0.167, and the 95% confidence interval is [0.016, 0.317], not including 0. It shows that career commitment has a significant mediating effect between calling and employee creativity.

**Figure 1:**
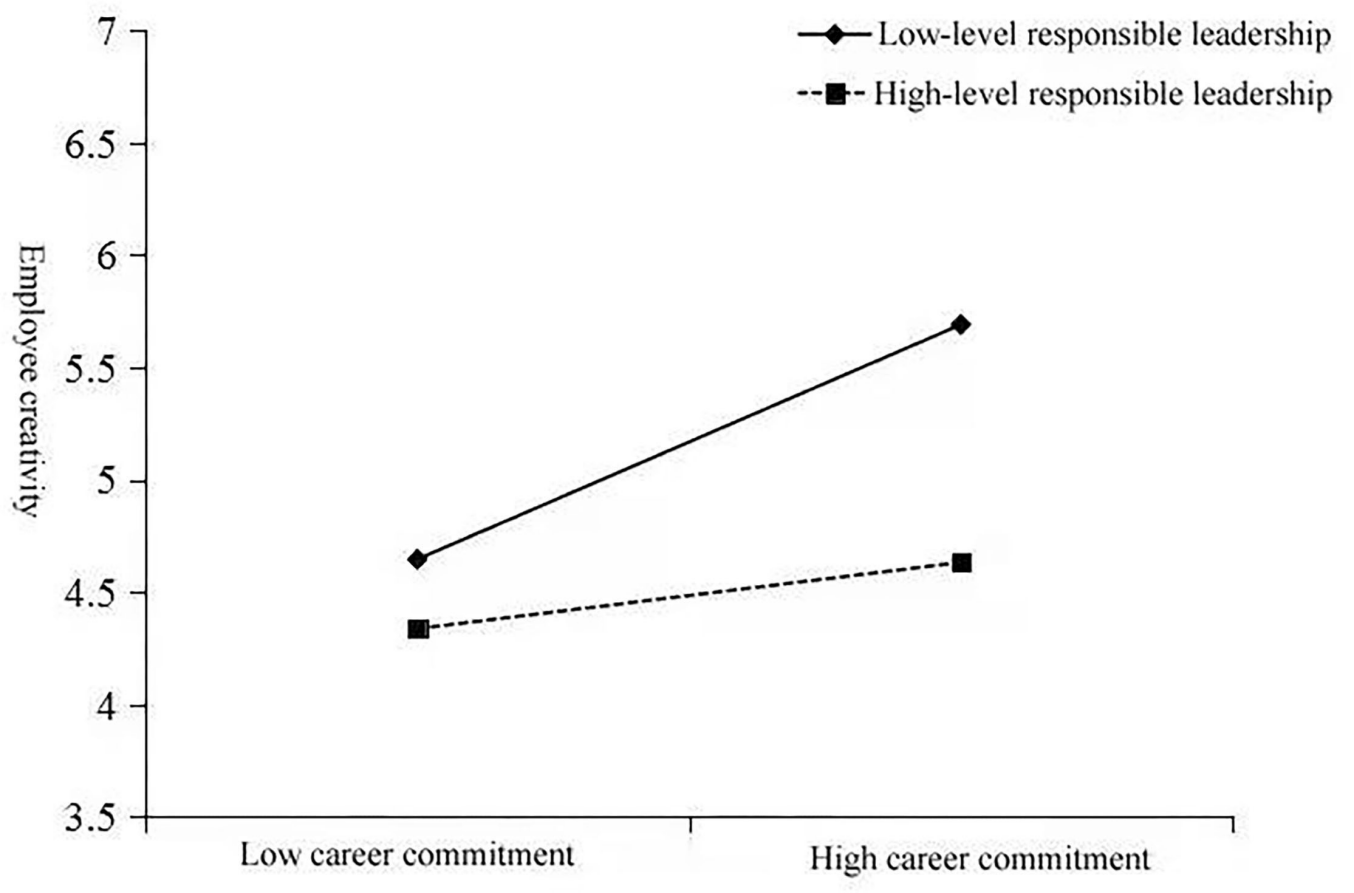
Interaction of career commitment and responsible leadership on employee creativity.

The regression results of the moderating effect are shown in Table 5. In Model 8, the interaction term of career commitment and responsible leadership has a significantly positive effect on employee creativity (β = 0.167, *p* < 0.01), indicating that responsible leadership strengthens the relationship between career commitment and employee creativity. Figure 1 illustrates that, unlike when responsible leadership is low, the effect of career commitment on career creativity is stronger when responsible leadership is high. Further simple slope analysis shows that when the level of responsible leadership is low, the regression slope of career commitment on creativity is 0.127, *p* < 0.05.When the level of responsible leadership is high, the regression slope of career commitment on creativity is 0.451, *p* < 0.001. Therefore, hypothesis 3 is supported.

**Table 5:** Moderation effect.

**Items**	**Employee**	**Model 7**	**Model 8**
	**creativity model 6**		
Age	0.089	0.072	0.049
Gender	−0.209^**^	−0.173	−0.187
Education level	−0.095	−0.092	−0.087
Working years	0.129	0.141	0.144
Career commitment		0.200^**^	0.250^**^
Responsible leadership		0.258^**^	0.256^**^
Career commitment × Responsible leadership			0.167^**^
R^2^ change	0.090	0.165	0.025
F value change	5.281^***^	23.379^***^	7.248^**^

** *p < 0.01*,

*** *p < 0.001*.

In addition, the SPSS PROCESS macro was used to further examine the moderated mediation effect. As shown in Table 6, in the context of low responsible leadership, the indirect effect of career commitment through the relationship between calling and creativity is non-significant, with a 95% confidence interval (CI) of [−0.167, 0.221]. In the context of high responsible leadership, career commitment has a significant mediating effect on the relationship between calling and employee creativity, with a 95% CI of [0.094, 0.449]. This suggests that the mediating effect of career commitment is affected by different levels of responsible leadership. That is, the higher the responsible leadership, the stronger the mediating effect of career commitment. Moreover, the index of moderated mediation shows that the indirect effect of career commitment on the relationship between calling and employee creativity is significant (95% CI: [0.012, 0.152]). The moderated mediation effect is further validated.

**Table 6:** Results of the moderated mediation effect.

**Mediator**	**Level of moderator**	**Indirect effect**	**SE**	**95% CI**	**Index**	**SE**	**95% CI**
	Mean-SD	0.043	0.099	[−0.167, 0.221]			
Career commitment	Mean	0.152	0.083	[−0.013, 0.315]	0.084	0.036	[0.012, 0.152]
	Mean+SD	0.261	0.092	[0.094, 0.449]			

### Implications

This study has some theoretical contributions. First, previous studies have explored the positive relationship between calling and work outcomes (e.g., Hirschi and Herrmann, 2012; Duffy and Dik, 2013), but only a few have linked calling to employee creativity. In today's business world, the internal and external environments of organizations are undergoing unprecedented changes. Employee creativity is the foundation of enterprise innovativeness, which is considered the most significant competitive advantage. Therefore, this study shows that calling plays an important role in stimulating employee creativity. Second, this study introduces career commitment as a mediator in the model. Although previous literature has identified a positive relationship between calling and career commitment (e.g., Duffy et al., 2011), we explore their effects on employee creativity, which can extend the domain of relevant theories. Third, this study examines the moderating effect of responsible leadership on the model. Previous studies on responsible leadership have mostly focused on its effect on work outcomes, such as job performance and organizational citizenship behavior, and have ignored the role of responsible leadership on employees' creative behavior. This study tests the role of responsible leadership in promoting employee creativity, which enriches the relevant theory and provides a new research direction for the future. In addition, this study finds that an individual's personal character and leadership style can interact to influence employee creativity. This provides a multi-angle and multi-level thread for future research in this area.

In terms of practical implications, this study shows that calling enhances career commitment and stimulates creative behavior. Therefore, to promote employees' innovativeness, employers should make efforts to improve employees' calling and career commitment. Specifically, managers should optimize the design of work tasks. Specific strategies include delegating authority to enhance employees' sense of calling. The sense of calling experienced by employees from these tasks can help them invest more time and energy in creating. Reasonable standards should be set around work objectives to meet employees' subjective value perception. Managers should also enrich the work content, expand work responsibilities, establish reasonable promotion channels, and strengthen work communication and contact with staff. These methods can make employees feel important in a team or organization, enhancing their self-efficacy. Moreover, in the process of building organizational culture, employers should emphasize organizational mission and vision and strive to seek the fit between organizational mission and employees' sense of calling. Lastly, the results show that responsible leadership can encourage employees to transfer calling and career commitment to substantive creative behavior, which provides a basis for leaders to stimulate employee creativity. Leaders should try to play the role of “responsible leadership” to give employees more autonomy and freedom and to actively convey trust to employees. This will give employees space for self-development and enable them to work in a more proactive manner to stimulate creativity in the organization. For employees, they should actively cultivate their sense of calling by identifying the meaning and value in their work. They should make efforts to improve work efficiency and enrich work content to make them feel a “sense of participation,” thereby enhancing their sense of calling.

## Limitation

This work has some limitations. First, this study used cross-sectional data; thus, dynamic changes in the focal variables were not observed. Therefore, we suggest that scholars conduct longitudinal research in the future to further verify the dynamic correlations between the variables to acquire more rigorous and robust conclusions. Second, the current study collected data through a questionnaire survey, which was not able to examine causal relationships. Future research should adopt an experimental method to verify the causal relationship between the variables. Third, we adopted self-report data in this study, which could have caused common method bias. Future studies should collect multi-source and even multi-level data to strengthen the validity of the conclusion. Lastly, only 218 valid samples were included in this study. Future research should replicate the study with a larger sample. In addition, based on the scope of this research, we focused on employees from Internet companies. In the future, researchers can collect data from other industries and make cross-industry comparisons to generate a more universal theory.

## Data Availability Statement

The raw data supporting the conclusions of this article will be made available by the authors, without undue reservation.

## Ethics Statement

Ethical review and approval was not required for the study on human participants in accordance with the local legislation and institutional requirements. The patients/participants provided their written informed consent to participate in this study.

## Author Contributions

JL designed the study, completed literature search, and paper writing. WC contributed to hypothesis development and the parts of Implications and Limitation. YR was in charge of data collection and analysis. All authors contributed to the article and approved the submitted version.

## Conflict of Interest

The authors declare that the research was conducted in the absence of any commercial or financial relationships that could be construed as a potential conflict of interest.

## Publisher's Note

All claims expressed in this article are solely those of the authors and do not necessarily represent those of their affiliated organizations, or those of the publisher, the editors and the reviewers. Any product that may be evaluated in this article, or claim that may be made by its manufacturer, is not guaranteed or endorsed by the publisher.
